# Increasing transparency of decision making in research practice: adding value or just more red tape?

**DOI:** 10.1007/s10654-025-01205-0

**Published:** 2025-02-24

**Authors:** Jenny T. van der Steen, Lex M. Bouter

**Affiliations:** 1 Department of Public Health and Primary Care, Leiden University Medical Center, Hippocratespad 21, Building 3, P.O. Box 9600, Leiden, 2300 RC The Netherlands; 2 Department of Primary and Community Care and Radboudumc Alzheimer Center, Radboud University Medical Center, Nijmegen, The Netherlands; 3 Cicely Saunders Institute, King’s College London, London, UK; 4 Department of Epidemiology and Data Science, Amsterdam Public Health Research Institute, Amsterdam University Medical Centers, Amsterdam, The Netherlands; 5 Department of Philosophy, Faculty of Humanities, Vrije Universiteit Amsterdam, Amsterdam, The Netherlands

**Keywords:** Research integrity, Research activities, Research methodology, Research protocol, Data reporting, Reporting guidelines

There is a broad consensus that a full study protocol needs to be written, and preferably be uploaded in a suitable repository, before embarking on data collection and data analysis. Nevertheless, when executing the study plan, scholars make decisions all the time. Decisions may be discussed, discussed and reported, or they happen unnoticed. In twelve months of fieldwork in two end-of-life care research groups, our ethnographer, PhD candidate van Drimmelen, witnessed “abundant instances of researcher discretion” despite detailed planning of the research [1]. Suppose this field of healthcare research was not, and these experienced research groups were not, unique in encountering multiple methodological and ethical challenges in research practice: what could be implications? Do we need to take steps to increase transparency of the research process? Would transparent reporting of these decisions add value in terms of an acceptable return on investment?

When reflecting on these questions, and essentially, on what it is to be a good scientist and what responsible research practices are, one can take three perspectives: normative, empirical and hermeneutical. The normative perspective formulates the principles and standards of research that are considered to be relevant, morally acceptable, and of good quality and integrity. Its operational aspect is spelled out in laws, regulations, codes of conduct and standard operating procedures, including reporting guidelines such as provided by the Equator Network [2]. Over the last decades, a normative emphasis on transparency and accountability has been substantiated in norms concerning open methods and open data. The empirical perspective entails investigating how actual decisions are made in research such as in ethnographic fieldwork, but also how actions influence validity, precision and reproducibility, e.g. by studying cumulation of biases in research practice [3].

The hermeneutic perspective is, for instance, used in (auto)biographies of famous researchers or in novels [4]. An understudied hermeneutical approach is provided through ethnographic research observing and interviewing scholars as they practice. This helps to understand what researchers actually believe and think ‘from the inside’ what it is to be a good scholar. Ethnography also offers a strong empirical perspective focussing on what scientists actually do such as in Latour’s classical studies on day-to-day laboratory life [5].

How should we interpret the observations made in van Drimmelen’s ethnographic fieldwork? The study showed that the researchers struggled in resolving ambiguity in their study protocols. Such ambiguity may of course emerge when a study protocol lacks sufficient detail. However, this also occurred when the protocol was too detailed, necessitating multiple adaptations in retrospect. As a result of this and from ambiguities in the research plan that became evident only when executing them, the development of the study protocol continued during its execution.

One could argue that findings such as a questionnaire that turns out to be more burdensome than expected for a subset of participants, necessitating adaptation, might have been prevented through pilot testing. However, this may have been unfeasible or undesirable as thorough feasibility studies come with costs. To what extent should we, to shift planning back to before the full study commences, pursue comprehensive pilot studies (to become ‘pre-studies’)−if achievable at all? Contingency planning, considering various scenarios to decide upon in advance, may help to the extent that researchers are aware of scenarios that may require future decision making. However, our ethnographer found that during the research, it was occasionally not clear to the researchers that they were making a decision, or they weren’t aware of having made one afterwards.

Checklists of decisions that might be necessary to make during data collection and data analysis may be helpful, as checklists have shown their worth in study design and reporting, and in peer review. In contrast, checklists have been abandoned in advance care planning research, because checklists to guide discussions and documentation on preferences for future (end-of-life) care have shown to be insufficiently effective in obtaining the preferred care. The preferences documented before a crisis in health emerges−in for example, advance directives or living wills−often lack detail which is needed to capture the exact scenario encountered later on [6, 7]. Therefore, recent conceptualizations of advance care planning revolve around ongoing person-centred conversations between patient and health care provider, adapted and documented as needed [8]. A checkbox approach to take potentially relevant future decisions in advance also fails in educating people on what might happen, and in increasing awareness of their values relevant to future decisions. In analogy to advance care planning in clinical practice, in research practice as witnessed by van Drimmelen, decisions made in advance as documented in the study protocol frequently need adaptation during data collection and analysis. Apparently, also in research a one-off protocolized or scripted tick-box approach does not suffice. Rather, ongoing conversations within the study team can increase awareness of new decisions that need to be made and expose underlying values at stake. Prompts during project team meetings in the form of open questions might help; for example, did we, or do we need to, decide on a deviation from the protocol? If so, why does it matter; does it affect quality or integrity of the research? Does it need a protocol revision or formal amendment now or are we merely to report it in a future publication?

Should we strive to document all deviations from the study protocol? This involves administrative burden and costs for which incentives are needed. Adding to administrative burden may be accepted if professionals feel that it purposefully aligns with intrinsic motivation and adds value to their practice [9]. In the end, the cost-benefit ratio of taking time to increase awareness of decisions made during data collection and data analysis, and to document what matters, might be more favourable than check-box approaches alone.

How to decide which research decisions matter? Suppose we take the 52 decisions observed by van Drimmelen and map them onto lists of items considered relevant for validity, precision and reproducibility [10] to start compiling an extensive checklist of all possible decisions during the conduct of research. Researchers can then select those that matter and should be reported. Such flexible choosing from an expanding meta-collection of potentially relevant items has improved the quality of implementation research [11, 12]. Benefits for the phase of study design included increased researchers’ awareness of the range of aspects that may be explored, and benefits for subsequent research phases included promoting of a shared language of those aspects [12] which is also relevant to reporting of research.

This brings us to the question whether there is value in expanding the current reporting guidelines with a set of optional awareness-raising items around decisions made during data collection and data analysis. However, if used in the reporting phase only, it may be too late to recall potentially relevant decisions made when conducting the research. Log-keeping of decisions, as is common practice in laboratories, may increase transparency in reporting healthcare research. A conversation with an independent researcher about which of the decisions matter most, may enhance its value, or it may allow for evaluating the quality of decisions made when executing the research, so as to make good research better. Further, reflecting on what is called positionality in the social sciences and non-financial conflict of interest in biomedicine may help researchers to critically review their decisions during the research process. By this we mean reflecting on strong societal, political or religious personal convictions that may induce bias. We definitely need more research to understand the effects of such interventions. We will need to evaluate potential upsides, downsides and unintended consequences [7] of monitoring researchers’ decisions, to answer the question whether they add value rather than red tape.

Education of responsible conduct of research may target decision making in daily research practice, to increase awareness of the decisions made during data collection and data analysis and to understand relevance of decisions for research quality and transparency of reporting. Novice researchers may understand research practice as the smooth, logical process which is reported in the organized, crisp and clear method section of scientific publications, which does not help in making them aware of relevant struggles and the decisions that need to be made in practice. They may be supported by regularly discussions of hypothetical or experienced struggles with peers, or also with experienced researchers in a safe environment.

Efforts in fostering responsible research practices should not only target researchers and research organizations offering relevant education and stimulate a suitable research culture, but should simultaneously target funding organizations, and scientific publishers [13]. Publishers should encourage reporting of deviations from the study protocol, with guidance that encourages researchers to reflect on decisions made during data collection and data analysis. Emphasising the value of, and interest in the complete methodological narrative of the study, may appeal to researchers’ intrinsic motivation to report on how they creatively optimized the research and went that extra mile despite encountering unforeseen situations in research practice. Nevertheless, researchers may have to strike a balance between underdetermination and overdetermination of study protocols and in adapting them during data collection and data analysis, and let others learn from how they did that.
